# A retrospective pilot study of the use of a new algorithm to improve quality control in bronchodilator studies

**DOI:** 10.3402/ecrj.v2.26949

**Published:** 2015-03-30

**Authors:** Charlotte L. Earle, Rhys Jefferies

**Affiliations:** 1 Aneurin Bevan University Health Board, St. Woolos Hospital, Newport, Wales, UK; 2 College of Human and Health Sciences, Swansea University, Wales, UK

**Keywords:** quality control, inhaler therapy, bronchodilator response, bronchodilation, asthma, mid-expiratory flow rates

## Abstract

Reversibility testing is used to identify a positive or negative response to bronchodilators. Results from a reversibility test can not only support a diagnosis of asthma but can alter a patient's treatment plan, so its clinical importance should not be understated. With multiple guidelines published classifying a ‘positive response’ it becomes unclear on how to categorise certain individuals. This study looks into the discrepancies between the guidelines, and introduces a new algorithm to help clinicians. This retrospective pilot study was completed across four hospitals in South Wales. Data were collected from a total of 117 patients referred for a reversibility study during November 2013 and April 2014. An algorithm was created to improve flow-volume loop (FVL) quality control when assessing airways bronchodilation in symptomatic patients. Each patient result was placed through four major reversibility guidelines [British Thoracic Society (BTS), National Institute for Clinical Excellence (NICE), Association for Respiratory Technology Physiologists (ARTP) and Global Lung Initiative (GLI)] and the new algorithm. When comparing published guidelines, 75% of patients would receive the same bronchodilator response decision, positive or negative, irrespective of the guideline followed. Variability between the numbers of positive responders in each guideline varied by up to 58%, with NICE found to give the least number of positive responses (7%), and BTS giving the greatest (65%). Using the new algorithm, over one third (38%) of patients required a repeat FVL, as baseline and/or post-bronchodilator FVLs did not meet the quality control specification. Further investigation is needed to establish the clinical impact of the new algorithm, and its approach to using the whole of the FVL in bronchodilator analysis; however, quality control during reversibility testing needs to be improved to ensure that bronchodilator responses are correctly identified.

Asthmatic patients are frequently referred to pulmonary function laboratories for reversibility studies to objectively assess bronchodilation. These patients typically present with wheeze, cough and chest tightness. However, a high proportion of these patients, despite reporting symptomatic benefit from bronchodilator therapy, do not meet the traditional spirometric criteria to determine bronchodilation.

The British Thoracic Society (BTS) Asthma guidelines (1) suggest a diagnosis of asthma is a high probability if the patient suffers with wheeze, breathlessness, chest tightness and/or cough, especially if these symptoms worsen on exercise, at night or early morning, and/or after exposure to allergens. Asthma is also highly probable if there is a family history, unexplained low forced expiratory volume in 1 s (FEV_1_) or peak expiratory flow (PEF), wheeze on auscultation, history of atopic disorder or unexplained elevated peripheral blood eosinophilia (1).

A bronchodilator response is regularly used to assess the degree of reversible airways obstruction, with many guidelines published to support a clinical diagnosis. Discrepancies exist between published reversibility guidelines regarding the definition of a positive bronchodilator response, i.e. some guidelines use a percentage increase whilst others suggest volume changes.

The aims of this study are:

- To create an algorithm to improve quality control during bronchodilator testing; ensuring that an increased FEV_1_ or PEF is due to bronchodilation rather than technical or effort changes
- To test whether patients with a ‘normal’ FEV_1_ and FEV_1_/forced vital capacity (FVC)% may bronchodilate according to low baseline mid-expiratory flow rates (MEF_25–75_)
- To establish a sub-group of patients who may benefit from an inhaler trial or provocation test

## Methods

Retrospective bronchodilator responses from 117 patients (m=49, f=68) were collected between November 2013 and April 2014, across four hospitals in South Wales: St Woolos Hospital (Newport), Nevill Hall Hospital (Abergavenny), Singleton Hospital (Swansea) and Morriston Hospital (Swansea).

A new algorithm, based around current guidelines, was constructed. This algorithm was designed to allow effective assessment of bronchodilation using the full flow-volume loop (FVL), without the influence of technique variability. By establishing well-defined parameters during baseline and post-bronchodilator spirometry a ‘grey’ area, between the values defining a significant bronchodilator change and the values defining intra-test repeatability of spirometry was identified, in the hope that this may add clinical value for further investigation in symptomatic patients.

**Fig. 1:**
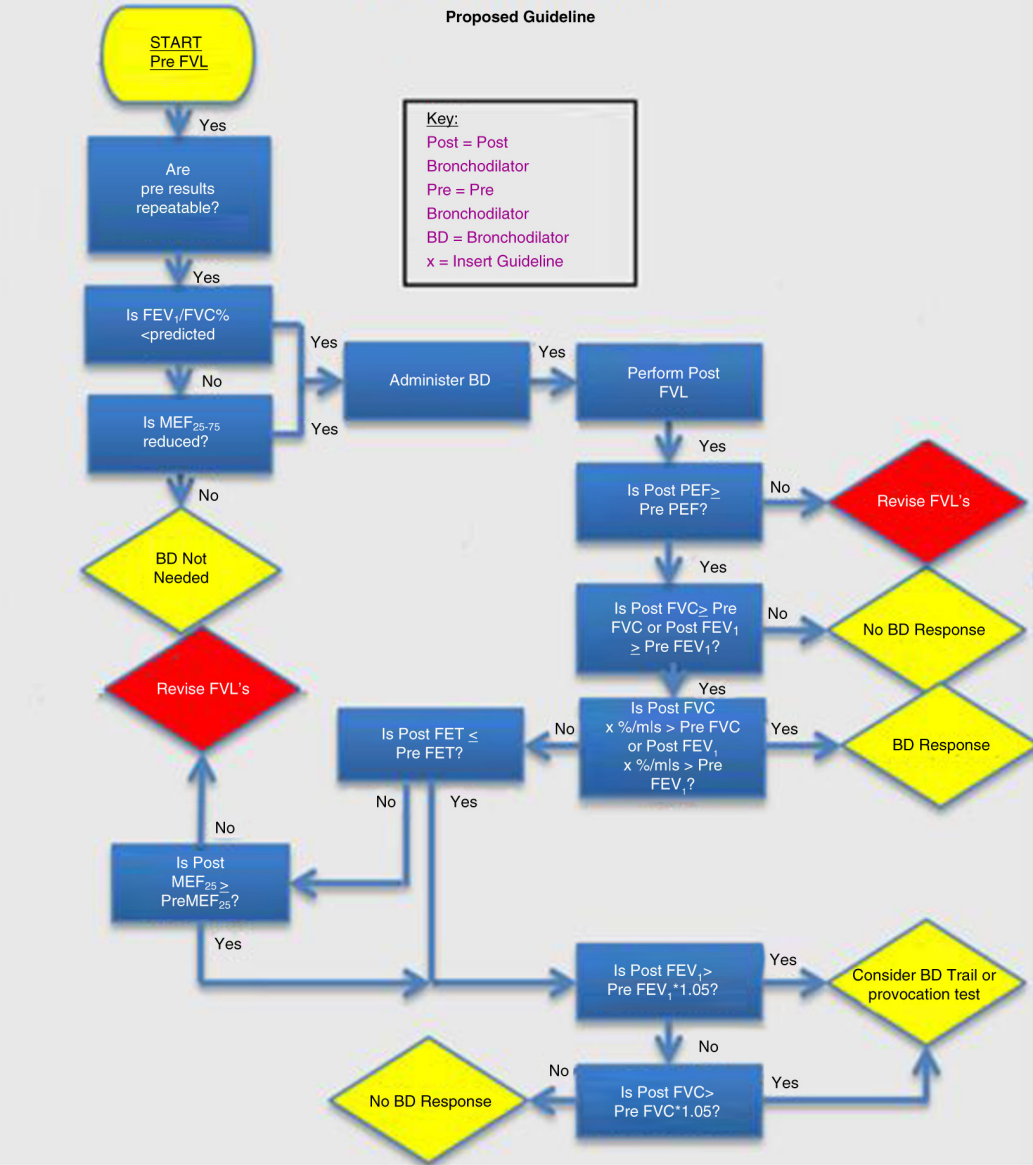
The new flow chart for assessing bronchodilator response. Guideline Descriptions 1. British Thoracic Society (BTS) (1): considered in this pilot study as >12% increase in FEV1 or PEF 2. European Respiratory Society (ERS)/Global Initiative for Chronic Obstructive Lung Disease (GOLD) (2): >12% increase in FEV1 and >200 ml 3. Association for Respiratory Technology Physiologists (ARTP) (3): >160 ml increase in FEV1 or >330 ml increase in FVC 4. National Institute for Clinical Excellence (NICE) (4): >400 ml increase in FEV1

Each result was passed through different accepted guidelines 1–5 (see additional material) to determine the percentage positive and negative response. We tested the BTS (1), European Respiratory Society (ERS)/Global Initiative for Chronic Obstructive Lung Disease (GOLD) (2), Association for Respiratory Technology Physiologists (ARTP) (3) and National Institute for Clinical Excellence (NICE) (4) guidelines. Each result was then passed through the new algorithm shown in Fig. 1 (5) substituting the BTS guideline for ‘x’.

Patients who experienced a degree of reversibility but not enough to classify them with having a positive response were categorised into a ‘consider provocation test/steroid inhaler trial’ group, indicating that further investigation may be necessary.

## Results

The BTS guideline has the highest percentage of positive responses (65%), whereas NICE only classify 7% of patients positive responders. ARTP and ERS lie in between; classifying positive responses in 48 and 36%, respectively.

When comparing published guidelines 1–5, 75% of patients would receive the same bronchodilator response decision, positive or negative, irrespective of the guideline followed. This leaves one quarter (25%) of patients with ambiguity in their bronchodilator response classification. It is possible that this group contain those identified with having poor quality control from following the new algorithm.

Figure 1 describes the new algorithm. The initial stages of the algorithm involve achieving quality, repeatable baseline FVLs to establish airways obstruction, either by a reduced FEV_1_/FVC ratio or MEF's, before administering the bronchodilator. The next stage of the algorithm allows the department to input their own reversibility criteria, depending on departmental protocol. This is followed by analysis of forced expiratory time (FET) and MEFs.

The final stage of the algorithm identifies patients whose post-FEV_1_ or FVC has not improved greater than the reproducibility criteria (i.e. 5%). It would be beneficial to re-assess these patients following an inhaler therapy trial or provocation test.

The new algorithm identifies an equal number of 30 (26%) patients, who respond positively and negatively. According to the new algorithm, two patients (2%) were not suitable for a BD test. In addition, a total of 44 patients (38%) were identified to require a repeat FVL, due to poor quality control, before a decision on bronchodilator reversibility can be made. Another 14 (12%) patients were recommended for an inhaler trial or provocation test.

This algorithm establishes stringent quality control criteria during reversibility testing, allowing accurate identification of patients who may benefit from inhaler therapy. It also prevents patients with atypical evidence of airways obstruction [reduced mid-expiratory flow rates (MEF_25-75_), and a forced expiratory volume in one second (FEV_1_)/forced vital capacity (FVC)>0.7)] being excluded from reversibility investigations. Additionally, this algorithm suggests whole FVL analysis: MEF_25-75_, PEF, FET, FEV_1_ and FVC, may be more effective in identifying patients that would benefit from an inhaler trial.

## Discussion

Improved quality control in reversibility testing is necessary to ensure bronchodilator responses are correctly identified. This will increase the sensitivity of results and help prevent inaccurate decisions. There are many irregularities amongst published guidelines regarding the definition of a positive bronchodilator response; these should be carefully considered when analysing bronchodilator results.

**Fig. 2:**
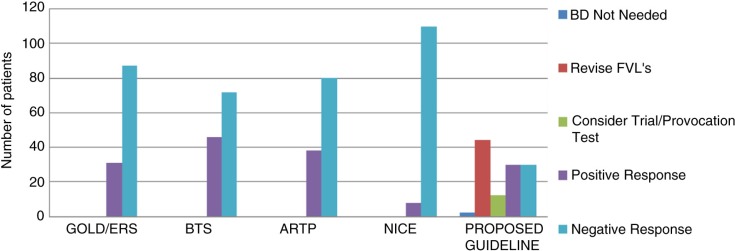
Discrepancies in the number of positive and negative bronchodilator responses according to the guideline used for interpretation. Results following the flow chart in Fig. 1 are also shown.

Figure 2 shows the number of patients that would be classified as having a positive and negative bronchodilator response depending on the guideline followed by the lung function department. There is good correlation between published guidelines (BTS, ERS/GOLD, ARTP and NICE) for results showing a definitive positive response (where there is a large percentage, or absolute volume, change in FEV_1_ or FVC) and those showing a definitive negative response (i.e. less than intra-test variation). Disparities exist where patients improve beyond intra-test variation limits, yet do not exceed the required guideline response (e.g. ERS 12%). The final column shows the distribution of patient responses should the algorithm be used. The algorithm gives equal numbers of positive and negative responses, with more positive responders than NICE. Although results from this algorithm cannot yet be compared with published guidelines, the quality control aspect identifies 38% of patients require a revised FVL before a truly accurate clinical decision can be made. Once quality control is improved, it is possible that some of these patients may fall into the provocation/inhaler trial group in the algorithm.

The algorithm in this pilot study was created to ensure that clinicians identify poor quality FVLs and accurately assess bronchodilator response. A rigid quality control procedure is essential to ensure that a bronchodilator response is correctly assigned.

We propose that FET should decrease upon bronchodilation, as airways resistance decreases, therefore, facilitating greater flow. In contrast, however, a larger FVC due to bronchodilation could also increase FET as airways collapse occurs later in expiration (reduced residual volume). To accommodate this, the post-FET should be equal to or less than pre-FET, unless the post-MEF_25_ is greater than pre-MEF_25_.

The use of MEFs in assessing bronchodilation in children with normal FEV_1_ was supported by a recent study (6). It has been suggested that MEF percent predicted should be considered when determining if a child should undergo a reversibility study (6). This paediatric study found MEFs to be more sensitive to bronchial changes than FEV_1_ measurements (6), a finding also supported in adults (7).

The algorithm guarantees that technique changes do not influence a clinician's decision, i.e. a reduced effort and PEF, as this may alleviate dynamic compression of the airways, and reduce the sensitivity of the FEV_1_. The bronchodilator response criteria used in lung function departments vary; therefore, there is the option for each department to enter their own criteria into the algorithm (substituting for ‘x’).

Further analysis of the group of patients with poor quality control as defined by this algorithm is necessary. It would also be useful to use the new algorithm prospectively, i.e. to identify patients who should be requested for a BD test. The new algorithm provides a systematic method for ensuring rigorous quality control when using FVLs to assess a bronchodilator response. To establish clinical significance, particularly in symptomatic patients who bronchodilate beyond baseline variability but less than that defined by the guideline used, further investigation is necessary.

This study suggests that a more rigorous quality control protocol will increase the sensitivity of using FVLs to support a diagnosis of asthma.
